# Effect of heat therapy compared with strength training on metabolic profile, heat shock response, inflammation, cardiovascular responses and microbiota in individuals with type 2 diabetes: study protocol of a randomized trial

**DOI:** 10.1590/1516-3180-2024.0040.R1.13082024

**Published:** 2025-06-02

**Authors:** Patrícia Martins Bock, Lucas Stahlhöfer Kowalewski, Layane Ramos Ayres, Mariana Kras Borges Russo, Gabriela Tomedi Leites, Andreza Francisco Martins, Álvaro Reischak de Oliveira, Mauricio Krause

**Affiliations:** I Department of Physiology, Graduate Program in Pharmacology and Therapeutics, Institute of Basic Health Sciences, Universidade Federal do Rio Grande do Sul (UFRGS), Porto Alegre (RS), Brazil.; II Laboratory of Inflammation, Metabolism and Exercise Research (LAPIMEX), Laboratory of Cellular Physiology, Department of Physiology, Institute of Basic Health Sciences, Universidade Federal do Rio Grande do Sul (UFRGS), Porto Alegre (RS), Brazil.; III Laboratory of Inflammation, Metabolism and Exercise Research (LAPIMEX), Laboratory of Cellular Physiology, Department of Physiology, Institute of Basic Health Sciences, Universidade Federal do Rio Grande do Sul (UFRGS), Porto Alegre (RS), Brazil; PhD student, School of Physiotherapy, Physical Education and Dance, Universidade Federal do Rio Grande do Sul (UFRGS), Porto Alegre (RS), Brazil.; IV Laboratory of Inflammation, Metabolism and Exercise Research (LAPIMEX) and Laboratory of Cellular Physiology, Department of Physiology, Institute of Basic Health Sciences, Universidade Federal do Rio Grande do Sul (UFRGS), Porto Alegre (RS), Brazil.; V Department of Physiotherapy, Universidade Federal de Ciências da Saúde de Porto Alegre (UFCSPA), Porto Alegre (RS), Brazil.; VI Department of Microbiology, Immunology, and Parasitology, Universidade Federal do Rio Grande do Sul (UFRGS), Porto Alegre (RS), Brazil.; VII School of Physiotherapy, Physical Education and Dance, Universidade Federal do Rio Grande do Sul (UFRGS), Porto Alegre (RS), Brazil.; VIII Laboratory of Inflammation, Metabolism and Exercise Research (LAPIMEX) and Laboratory of Cellular Physiology, Department of Physiology, Institute of Basic Health Sciences, Universidade Federal do Rio Grande do Sul (UFRGS), Porto Alegre (RS), Brazil; Adjunct Professor, School of Physiotherapy, Physical Education, and Dance, Universidade Federal do Rio Grande do Sul (UFRGS), Porto Alegre (RS), Brazil.

**Keywords:** Gastrointestinal microbiome, Heat-shock proteins, Exercise, Glycemic control, Gut microbiota, Inflammatory markers, Glucose control

## Abstract

**BACKGROUND:**

Interventions capable of modulating the heat shock response (HSR), such as physical exercise and heat therapy (HT), are potential therapeutic strategies for the prevention and treatment of diabetes.

**OBJECTIVE:**

This study aims to evaluate the effects of resistance training (RT) and HT on HbA1c levels, metabolic and inflammatory profiles, gut microbiota, and HSR in patients with type 2 diabetes mellitus (T2DM).

**DESIGN AND SETTING:**

A randomized, double-blind, parallel clinical trial will be conducted for 12 weeks in southern Brazil.

**METHODS:**

Participants with T2DM will be randomized into control (any intervention), RT, or HT groups. In the RT group, participants will perform supervised exercise, and the HT group will undergo whole-body heat treatment in an environmental chamber initially set at 55.0°C, both on three non-consecutive days of the week (60 min). Blood samples will be collected before and after 12 weeks of treatment. Heat shock response, body composition and physical fitness, glycemic control, lipid profile, gut microbiota composition and diversity, inflammatory markers, and flow-mediated dilation will be evaluated.

**CONCLUSION:**

Since the HSR response is decreased in individuals with diabetes, we believe that improving the HSR may be important in preventing chronic complications associated with T2DM. This randomized clinical trial will determine the efficacy of HT compared to RT in improving HSR when combined with conventional therapy in individuals with T2DM. Multiple HT and RT effects may contribute to a lower mortality risk in these individuals.

**CLINICAL TRIAL REGISTRATION:**

Unique Identifier NCT05847075. https://clinicaltrials.gov/study/NCT05847075.

## INTRODUCTION

An estimated 10.5% of all adults aged 20–79 years worldwide have diabetes, and approximately one-third of all deaths in people under the age of 60is attributed todiabetes.^1^ The cost of care for diabetes and the associated diseases is very high, but maintaining an individual with diabetes at low or even moderate risk of macro-and microvascular disease is a fraction of what is currently being spent on acute complications.^2^For this reason, the search for different strategies to prevent the onset of complications is essential.

One of the main characteristics found in type 2 diabetes mellitus (T2DM), as well as in obese and insulin-resistant individuals, is the chronic presence of low-grade inflammation, which may be related to the expansion of adipose tissue and also to the modification of the gut microbiota profile (gut dysbiosis).^3^ Lipopolysaccharide (LPS) produced by gram-negative bacteria cells may induce metabolic endotoxemia and stimulate systemic inflammation, which play a key role in insulin resistance.^4^ One of the important responses involved in the resolution of inflammation is the heat shock response (HSR), mediated by the action of 72 kDa heat shock protein (HSP72), whose expression is induced by a wide range of cellular stressors, such as heat, metabolic deprivation, redox imbalance and physical exercise.^5^

Dietary, surgical, and pharmacological interventions can alter the gut microbiota of patients with diabetes, leading to an increase in the gram-positive population compared to the gram-negative bacterial population.^6^ However, few studies have evaluated the effects of physical exercise on microbiota,^7-10^ and the precise contributions of physical exercise, including those related to metabolic endotoxemia in diabetes, remain to be investigated, especially in resistance training. In the context of inflammation, HSP72 is produced in the intracellular environment in response to stressful situations, and has an anti-inflammatory effect, blocking the activation of the main nuclear transcription factor related to inflammation, the nuclear factorkappaB (NF-κB).^11,12^ However, in conditions of chronic inflammation and insulin resistance, such as diabetes, the ability of cells to activate the HSR is compromised, causing cellular dysfunction, perpetuation of inflammation, and increased risk of complications in diabetes.^13^

Our group recently demonstrated that HSR is blocked in states of insulin resistance (IR), such as diabetes and aging, but can be partially restored by resistance training.^14^ Although physical exercise is a very efficient non-pharmacological tool for improving metabolic functions,^15^some people have difficulties in performing physical exercise.^16^ Therefore, an alternative therapy for special populations, which can induce an increase in HSP70 and other effects similar to those of exercise, is heat therapy (HT).^17^ In this sense, interventions capable of modulating the content of HSP72, such as physical exercise and HT, are candidates for the prevention and treatment of insulin resistance and diabetes.

Significantly, new evidence indicates that HT improves glycemic control in healthy individuals and individuals with T2DM by reducing fasting plasma insulin and increasing insulin sensitivity.^18^ A recent systematic review and meta-analysis found that HT can benefit people with metabolic diseases.^19^ However, only a few studies were included. Further randomized controlled trials with longer intervention and follow-up periods are needed to confirm the beneficial effects of passive heat therapy, which justifies the importance of our protocol in testing the potential benefits of HT for this population.

## OBJECTIVE

Due to few well-conducted clinical trials, we will perform a randomized, three arm, double-blind, parallel study, aiming to compare the properties of resistance training (RT) and HT in type 2 diabetes mellitus (T2DM). We hypothesize that RT and HT will reduce HbA1c levels, improve metabolic and inflammatory profiles, and attenuate gut dysbiosis and the HSR.

## METHODS

### Study design and setting

This prospective, randomized, double-blind, parallel, non-inferiority clinical trial will be conducted at the Universidade Federal do Rio Grande do Sul, a university in southern Brazil. The study protocol adheres to the SPIRIT recommendations.^20^

### Eligibility criteria

Participants will be included if they have type 2 diabetes (previously diagnosed by their personal physicians), were between 18-65 years old, with HbA1c levels between 7.0% and 10%, are sedentary non-smokers, body mass index (BMI) between 18.5-39.9 kg/m^2^, with controlled blood pressure, and no use of insulin. Participants will be excluded if they are pregnant or breastfeeding; report treatment with antibiotics or anti-inflammatory drugs within four weeks; report a history of myocardial infarction, cardiac illness, vascular disease, stroke, or any condition that would prevent them from engaging in an exercise study; or if they had already been engaged in two or more planned and structured exercise sessions per week (in the last six months).

### Ethics approval and consent to participate

The study strictly follows protocols regarding informed consent forms, confidentiality, and anonymity. An informed consent form with guidelines, project objectives, and a description of procedures, possible risks, and benefits will be provided. Participants will also be informed that participation is voluntary and that they can withdraw from the study at any time. This study will be conducted in accordance with the Declaration of Helsinki and was approved by the facility’s institutional review board on February 7, 2023 (approval number: 38626420.6.0000.5347) and registered in the Clinical Trials Database (NCT05847075). All files containing the participants’ data will be stored in a secure password-protected databasethat will be accessed only by authorized members of the research team.

### Allocation

Participants will be randomly assigned to groups according to a sequence of computer-generated random numbers. This sequence will be obtained in Microsoft Excel (Redmond, Washington, United States) in a 1:1 ratio, with blocks of eight or six participants to complete the total number calculated. A sealed envelope containing the allocation code will be opened at the beginning of the treatment in the presence of the participant according to the order of entry into the study.

Owing to the nature of the interventions, the researcher conducting the exercise sessions and participants will not be blinded. To ensure assessor masking, the participants will be asked to omit their assigned groups and not talk about their interventions during the outcome evaluation sessions. In case of unintentional unblinding for any reason, the researcher will notify the principal researcher.

### Interventions

Volunteers who met the inclusion criteria will be guided through the study and asked to sign a written informed consent form before enrollment in the trial. The participants will be randomized into the following intervention groups: control, RT or HT. In the control group, participants will not receive any intervention. In the intervention group RT, participants will perform supervised exercises in a gymnasium on three non-consecutive days of the week for a total of 12 weeks. Each session will last approximately 60 min and consist of warm-up, resistance training, and cool-down. All sessions will be conducted by qualified sports and exercise scientists.

The RT program will consist of a combination of upper- and lower-body exercises using gymnasium equipment, free weights, and body weight (functional exercises) as the primary resistance. The 12 weeks RT will be divided into three mesocycles of four weeks each (please see details of sets, repetitions, and intervals in Table 1). The exercises will include leg press, knee extension, leg curl, biceps curl, triceps extension, lat pulldown, shoulder press, bench press, and abdominal crunch exercises. Before the start of the training period, the participants completed a familiarization session to practice the exercises they would perform further during the training program when the exercise load was individually tested. Resistance training was performed using two–three sets per exercise at intensities between 12 and 15 maximum repetitions (the heaviest possible weight to perform 12–15 repetitions), as shown in Table 1. The intensity of the functional exercises was controlled using the OMNI scale,^21^ with each session lasting 60 min.

**Table 1:** Resistance training program

Mesocycle	Weeks	Repetitions/load	Number of sets	Interval between sets
**1**	1–4	12/15	2	1 min
**2**	5–8	12/15	3	1 min
**3**	9–12	10/12	3	1 min and 30 s

**Figure 1:**
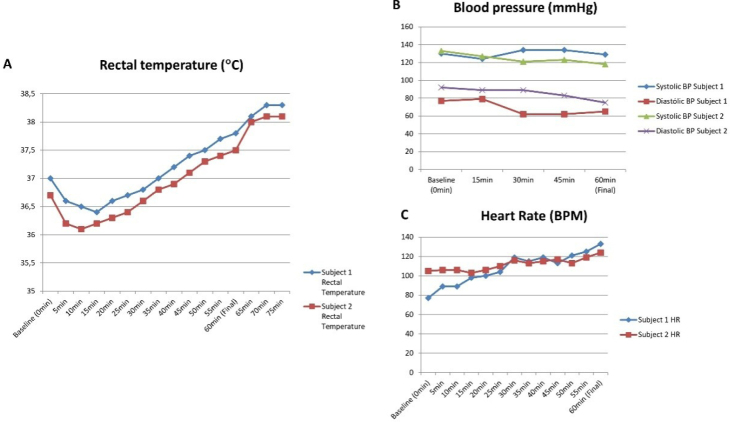
Heat therapy pilot testing.

Participants in the HT group will undertake whole body heat treatment according to previous methods.^22^ Participants will be housed in an environmental chamber initially set at 55.0°C on three non-consecutive days of the week. Each session will last 60 min. During the sessions, the rectal temperature, heart rate (HR), blood pressure, and heat discomfort will be constantly measured. According to pilot testing, this protocol induces an approximate 1.2°C raise in the individual’s core temperature and remains increased after the session for an additional 15 min, before it starts to drop (Figure 1a). The HR increases and blood pressure, particularly diastolic pressure, decreases during the session (Figure 1b and Figure 1c, respectively). The core temperature initially decreases (10-15 minutes after the beginning), probably owing to rapid skin vasodilation and a blood flow shift from the center to the periphery. After approximately 20 min in the chamber, the core temperature starts to rise, as expected. The characteristics of the participants from the preliminary study are shown in Table 2.

Physiological responses of subjects following 60 min in a55^°^C environmental chamber; A) rectal temperature; B) systolic and diastolic blood pressure (BP); C) heart rate (beats per minute (BPM).

**Table 2:** Pilot subjects characteristics

	Subject 1 (Healthy)	Subject 2 (Diabetic)#
Age (years)	42	60
Body Mass (kg)	63.3	88
BMI (kg/m^2^)	23.7	29.7
Glycaemia (mg/dL)	85	103

Both male subjects weretreated with metformin (Glifage) and empagliflozin (Jardiance).

BMI = body mass index.

### Body temperature assessment and thermal sensation

To measure the rectal temperature, a flexible thermometer (RET-1 Physitemp Instruments, Clifton, New Jersey, United States of America) will be inserted 10-12 cm beyond the anal sphincter (self-inserted by the participant following the researcher’s instructions). Skin thermometers will measure the skin temperature on the upper back, arm, and leg (left hemibody) (SST1, Physitemp). The environmental chamber will be set at 55.0°C. Participants’ temperatures will be continuously monitored and recorded every 5 min throughout the session. If a participant reaches a rectal temperature > 39.0°C and presents symptoms such as nausea, disorientation, tiredness, abdominal pain, or headache, the session will be stopped. In addition to temperature measurements, a perceptual scale was used to identify thermal sensation and thermal comfort. To this end, participants will be asked about their thermal sensation (9-point scale from “very cold” to “very hot”) and thermal comfort (6-point scale from “very comfortable” to “very uncomfortable”) every 10 min during the session.^23^

### Strategies for trial retention

Participants will be asked to monitor adverse events once a week during face-to-face visits. Moreover, phone calls or text messages will be used to remind participants of their scheduled or missed visits.

### Study outcomes

Outcomes will be evaluated at baseline and at the end of the study (12^th^ week). Outcome assessors and data analysts will be blinded to the intervention assignments. The primary outcome will be HSR.

A set of secondary clinical outcomes will include:

1. Body composition and physical fitness (anthropometric measurements, anthropometric and visceral adipose tissue measurements, dual-energy X-ray absorptiometry scan, peak VO_2_(peak oxygen uptake), basal metabolic rate)
2. Glycemic control (fasting glucose, insulin plasma levels, insulin resistance);
3. Lipid profiles (total, LDL, and HDL-cholesterol cholesterol and triglyceride levels)
4. Gut microbiota composition and diversity;
5. Plasma LPS concentration;
6. Inflammatory cell signaling in peripheral blood mononuclear cells (PBMC):NF-κB; toll like receptor (TLR) 2 e 4, TIR-domain-containing adapter-inducing interferon-β (TRIF), inhibitory-κB kinase (IKK)i/ε, e myeloid differentiation primary response 88 (MyD88);
7. Plasma LPS and PBMC inflammatory response to LPS;
8. Inflammatory/anti-inflammatory markers:C-reactive protein level, tumor necrosis factor(TNF)-α, interleukin (IL)1β, IL-4, IL-6, IL-10, HSP72, adiponectin and leptin);
9. Flow mediated dilation (FMD).

### Recruitment and participant timeline

**Figure 2:**
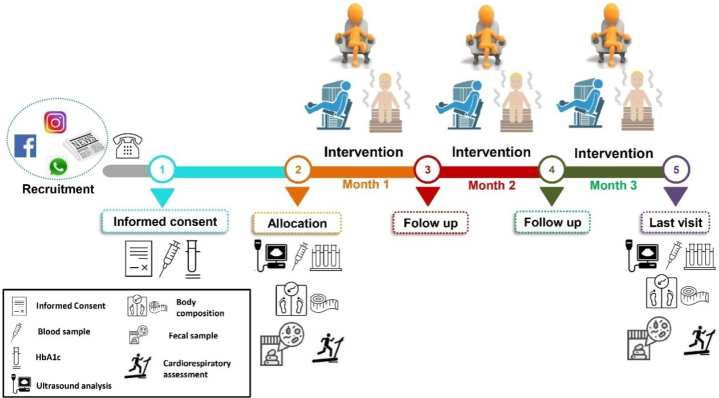
Flow of the study design.

The recruitment period for this study will be from January 2024 to December 2025. Participants will be invited to participate in the study through announcements in newspapers, electronic media, and social media. Figure 2 illustrates the study design.

1) Presentation of the study, signing of the informed consent form, and collection of HbA1c; 2) start of the study, data collection, anthropometric measurements, and collection of blood and feces; 3) monthly intervention; 4) monthly intervention; and 5) end of the study, data collection, anthropometric measurements, and collection of blood and feces.

### Adherence

Adherence to an exercise program was defined as meeting > 80% of the recommended or prescribed exercise doses. Any participant who demonstrates training protocol adherence of 80%, or attends more than 36 sessions, will be considered adherent. Participants will be classified as non-adherent or partially adherent if their adherence is < 80%.

### Statistical considerations

To assess the effect of exercise on plasma HSP levels between the groups post-intervention, we assumed 80% power, 5% significance level, and an effect size (Cohen’s F) of 0.4. We estimated a dropout rate of 10%, and the calculated total number of patients was 22 in each group.^24^

The randomly assigned participants’ data will be analyzed according to the intention-to-treat principle as the primary choice and the per-protocol principle, with treatment, time, and treatment-time interaction registered as fixed effects. We will qualitatively document the reasons for, and details of, each withdrawal case. The normality of the data will be tested using the Shapiro-Wilk test. Mean and standard deviation will be used to describe parametric continuous variables, with median and interquartile range for non-parametric variables, and absolute and relative frequencies for categorical variables. Analysis of variance (ANOVA) will be used to compare the final outcomes. Treatment effects (group, time, and group interaction) will be estimated using Generalized Estimation Equations (GEE), followed by the Bonferroni post-hoc test (P < 0.05). Multiple linear regression analysis (backward method) will be performed to investigate the impact of routine pharmacotherapy, sex, and age on the primary and secondary outcomes. SPSS Professional software (version 20.0; IBM Corp., Armonk, New York, United States) will be used for all analyses.

### Data collection and management

A questionnaire will be employed to obtain the sociodemographic and clinical characteristics of the participants, including age, sex, marital status, schooling level, diabetes duration, family history of diabetes, use of antimicrobials in the last 30 days, and self-reported physical activity. T2DM drugs will be recorded at baseline and end of the study period.

Anthropometric measures, including body weight, height, calculated body mass index (kg/m^2^), waist circumference, and hip circumference changes will be recorded at the first and last visits. Weight will be measured using a digitally calibrated balance with a maximum capacity of 150 kg and a precision of 100 g. The height will be determined by a vertical statometer attached to the balance with a capacity of 2 m/0.1 cm (Urano, Canoas, Brazil). Waist and hip circumferences will be measured using an inelastic metric tape (2 m/0.1 cm). The total corporal composition will be evaluated using dual-energy X-ray absorptiometry (DEXA scan, Lunar iDXA, GE Healthcare, Buckinghamshire, United Kingdom).

After this evaluation, individuals will perform a self-limited maximal exercise test on a treadmill to determine peak oxygen uptake (VO_2_ peak) and peak HR, supervised by a team of experienced exercise physiologists. The test will be carried out according to the Bruce protocol.^25^ Oxygen uptake (VO_2_) and carbon dioxide production (VCO_2_) were determined using a breath-by-breath computerized gas exchange system and analyzed using a 20-second averaging signal. The basal metabolic rate will be analyzed in a fasting state (Quark CPET, Cosmed, Italy).

At the first and last visits to the laboratory, blood samples will be collected and centrifuged at 1,000 × g for 10 min to separate the plasma, which will be stored for further analysis of biochemical parameters. The total glucose, C-reactive protein, HDL cholesterol, HbA1c, and triglyceride levels will be determined using an automated Cobas C111 system (Roche Diagnostics, Basel, Switzerland). LDL-cholesterol will be calculated by the Fried Ewald equation.^26^ Insulin resistance was calculated using the Homeostatic Model Assessment (HOMA-IR) method based on insulin and fasting glucose levels.^27^

To analyze the gut microbiota, genomic DNA will be extracted from 0.25 g of homogenized fecal samples using the QIAamp PowerFecal Pro DNA Kit (Qiagen, United States), according to the manufacturer’s instructions. The quality of the DNA will be evaluated by agarose gel electrophoresis and purity will be assessed by the 260/280 nm and 260/230 nm ratios measured using a NanoDrop 1000 instrument (Thermo Fisher Scientific, Waltham, MA, United States). DNA concentration will be quantified using Qubit dsDNA Reagent (Molecular Probes, Thermo Fisher Scientific Division, Eugene, OR, United States). The V3-V4 hypervariable region of the 16S rRNA gene will be amplified and sequenced using Illumina MiSeq V3 kit (2 × 300 cicles). The preprocessing and follow analysis will be conducted in R within the RStudio platform, using software package DADA2 for taxonomic assignment (with the SILVA ribosomal RNA gene database).^28-31^

For inflammatory biomarkers and plasma LPS levels, patientblood will be centrifuged at 1,000 g for 10 minutes and serum will be frozen (−80°C) for further analysis by the enzyme-linked immunosorbent assay (ELISA) technique, using commercial kits for: LPS, TNF-α, IL-1, IL-4, IL-5, IL-6, IL-10, and IL-13 (Human ELISA Kits, Sigma-Aldrich, St. Louis, MI, United States).

Considering the importance of HSR in stress adaptation, we will test the capacity of leukocytes (a major source of circulating HSP72 and representative of the immune cell stress response) to release HSP72 under heat stress conditions (a physiological and expected response in healthy cells). We will use this strategy to compare whether different interventions can improve HSR in patients with diabetes. Briefly, after harvesting, whole blood will be immediately incubated at two different temperatures: 37°C (control) and 42°C (heat stressed) for 2 h in a water bath. After incubation, the total blood will be centrifuged to isolate the plasma. Plasma will be used for the analysis of extracellular HSP72.^32^ A highly sensitive, enzyme-linked immunosorbent assay (EIA) method (EKS-715 Stressgen, Victoria, Canada) will be used to quantify the levels of plasma HSP72 protein, as previously described.^33^ Inflammatory cell signaling in PBMC (NF-κB; TLR2 e 4, TRIF, IKKi/IKKε, and MyD88) will be analyzed by western blot, as previously described.^34^

### Flow mediated dilation

Brachial artery FMD in response to hyperemia will be performed using an ultrasound device (Toshiba model Nemio XG, Japan) as an indirect measurement of endothelial function, adapted to the current guidelines.^35^

### Hydration protocol

On the day prior to heat exposure, participants will be instructed to drink 12 mL/kg^-1^ of water (in addition to usual liquid consumption). During the morning of the experimental session, the participants will be instructed to consume 6 mL/kg^-1^ of water, in addition to the usual liquid consumption, to ensure a state of euhydration. Prior to entering the environmental chamber, a urine collection will be performed to verify the state of hydration, using the specific gravity measured through a refractometer^36,37^ and assessment of the urine color through the Armstrong urine color chart.^38^ A urine specific gravity cutoff of 1.25 will be applied. For higher values, the participants will drink 200–250 mL of water and wait 30 min before heat exposure to ensure that they are normally hydrated before the session.

### Safety and adverse events

Participants will be under continuous health professional control for at least 30 min after the intervention to prevent adverse events. The following mild and moderate adverse events can occur. Exercise protocols, particularly during maximum effort, can cause discomfort (nausea and muscle soreness). Heat therapy has been shown to be safe for most people.^17^ However, for some participants, it is possible that the higher temperature caused thermal discomfort. Cardiovascular changes, such as increased heart rate and blood pressure, are expected and are not harmful. Mild dehydration may be observed after completion of the protocol and will be attenuated by proper hydration.

## DISCUSSION

Inflammation markers are increased in individuals with diabetes.^39^ Strength training has been shown to be a highly efficient therapeutic practice in the prevention and treatment of diabetes,^15,40^as it is capable of manage systemic inflammation by decreasing CRP levels, with inconclusive evidence for other inflammatory biomarkers.^41^ Furthermore RT leads to subcutaneous abdominal adipose tissue reduction^42^ and improves HSR,^14^ which could improve T2DM control and prevent chronic complications.

The first study evaluating the effects of HT in diabetes was published by Hooper et al. in 1999, and its primarily resulted in glycemia, HbA1c and body weight decreases.^43^ Remarkably, there are few studies analyzing HT and its effects on diabetes metabolic control in humans, and the analyzed trials in a systematic review had methodological limitations that preclude the use of therapy in clinical practice, such as small sample size, observational study design, and short-term treatment.^19^ Recently, a cross-sectional study found that habitual hot-tub bathing was associated with slight improvements in HbA1c and body mass index.^44^In contrast, a randomized crossover trial showed that 90 min of hot water immersion did not improve insulin sensitivity, markers of micro- and macrovascular functions, or circulating and intracellular HSP70 concentrations.^45^ There is a lack of consistent data in the literature, and, furthermore, the possible mechanisms by which benefits can be obtained remain unknown.

Since the HSR response is decreased in individuals with diabetes,^13^ we believe that improving the HSR may be important in preventing chronic complications associated with T2DM. This randomized clinical trial will determine the efficacy of HT compared to RT in improving HR when combined with conventional therapy in individuals with T2DM. Multiple HT and RT effects may contribute to a lower mortality risk in these individuals. Our study is the first to evaluate the efficacy of HT and RT in glucose, lipid, and inflammatory profiles, microbiota, and HR measurements in individuals with T2DM. Moreover, we will maintain the experimental protocol for 12 weeks, which is the time necessary to obtain changes in the main parameter of diabetes control, HbA1c.

## CONCLUSION

Owing to its multiple effects, HT may prevent the comorbidities commonly associated with chronic hyperglycemia. The use of HT as an adjuvant treatment may improve the quality of life of individuals with T2DM, delay the onset of comorbidities, and reduce health costs, which are essential to prevent primary risk factors. This information will have immediate applicability in the promotion of public health.
